# The 5:2 Diet Reduces Risk Factors for Cardiovascular Disease in Subjects with and Without Type 2 Diabetes—A Non-Randomized Controlled Trial

**DOI:** 10.3390/ijms27146460

**Published:** 2026-07-21

**Authors:** Anton Hellberg, Michaela L. Sundqvist, Angelica Lindén Hirschberg, Sergiu-Bogdan Catrina, Ingeborg Eriksson, Neda Rajamand Ekberg, Kerstin Brismar

**Affiliations:** 1 Department of Women’s and Children’s Health, Karolinska Institutet, 171 77 Stockholm, Sweden; angelica.hirschberg.linden@ki.se; 2 Department of Physiology, Nutrition and Biomechanics, The Swedish School of Sport and Health Sciences, 114 33 Stockholm, Sweden; michaela.sundqvist@gih.se; 3 Department of Gynecology and Reproductive Medicine, Karolinska University Hospital, 171 76 Stockholm, Sweden; 4 Department of Molecular Medicine and Surgery, Karolinska Institutet, 171 76 Stockholm, Sweden; sergiu.catrina@ki.se (S.-B.C.); erikssoningeborg@gmail.com (I.E.); neda.ekberg@ki.se (N.R.E.); kerstin.brismar@ki.se (K.B.); 5 Centre for Diabetes, Academic Specialist Centre, 113 65 Stockholm, Sweden; 6 Department of Endocrinology, Karolinska University Hospital, 171 76 Stockholm, Sweden

**Keywords:** intermittent fasting, 5:2 diet, type 2 diabetes, CVD

## Abstract

This is a secondary analysis of a non-randomized controlled six-month trial concerning the effects of the 5:2 diet on cardiovascular risk factors (blood pressure, blood lipids, adipokines and inflammatory markers) in overweight or obese individuals with and without type 2 diabetes (T2D). Ninety-seven individuals were recruited, 35 with T2D and 62 without (controls). Ninety-three completed six months of the 5:2 diet, consisting of 2 days of fasting (500 kilocalories (kcal) for women and 600 kcal for men) and five days of their regular diet. A total of 82 participants (31 with T2D and 51 controls) attended the 12-month follow-up. After the six-month intervention, both groups lost body weight (−6.3% in the T2D group and −5.0% among controls). There were significant improvements in waist circumference, blood pressure, high-density lipoprotein cholesterol (HDLchol), low-density lipoprotein cholesterol (LDLchol) and serum levels of adiponectin. In addition, participants with T2D had decreased alanine transferase (ALT) and high-sensitive C-reactive protein (hsCRP), which were significantly different from the control group. The control group had significant decreases in triglycerides and leptin concentrations. At the 12-month follow-up, improvements remained for HDLchol and adiponectin in both groups. In addition, the T2D group had significantly decreased hsCRP and leptin concentrations and the control group showed a significant decrease in ALT. In conclusion, a six-month 5:2 diet resulted in significant improvements in cardiovascular risk factors in overweight or obese participants with and without T2D, with several improvements persisting at 12 months. Individuals with T2D appeared to gain somewhat greater metabolic benefits from the intervention than the control group.

## 1. Introduction

Overweight and obesity are increasing in prevalence globally [1]. Increased body mass index (BMI) is a risk factor for type 2 diabetes (T2D) as well as for cardiovascular disease (CVD) and liver steatosis [2,3]. T2D is independently intertwined with CVD [4,5]. The link between obesity and CVD is partially explained by the fact that visceral obesity and ectopic fat are associated with insulin resistance (IR), which is compensated by hyperinsulinemia to maintain normoglycemia. The hyperinsulinemia and IR lead to increased arterial stiffness, higher blood pressure and dyslipidemia, all constituting the metabolic syndrome with increased risk of both T2D and CVD [6].

Adipokines, such as adiponectin and leptin produced by adipose tissue, have been suggested to be the link between obesity, T2D and CVD through their effects on satiety, lipogenesis and insulin sensitivity [7,8,9]. Leptin, a key regulator of energy homeostasis, has disturbed regulation in obesity due to leptin resistance in the hypothalamus [10]. Adiponectin correlates inversely with IR, dyslipidemia, CVD and hypertension [11,12]. In addition, low levels of adiponectin predict future T2D in women [13].

Caloric restriction (CR) has traditionally been achieved by continuous energy restriction to reduce body weight and improve CVD risk factors in obese individuals with and without T2D. Continuous CR also improves glycemic control in T2D [14,15,16] and adiponectin, insulin and leptin resistance in obese individuals [17,18,19]. However, this dietary approach is often unsuccessful as a long-term intervention due to poor compliance [20]. In addition, weight-loss surgery and pharmacological therapies are associated with high costs and side effects [21,22,23]. Glucagon-like peptide 1 receptor agonists (GLP-1RA) have been shown to elicit a weight loss of at least 10% [24,25] but will most probably not be available to most individuals for long-term use [26].

In recent years, intermittent fasting (IF), a diet alternating between a period of fasting and non-fasting, has gained popularity [27]. It has been suggested that IF leads to the same beneficial effects as traditional continuous CR, but with better long-term compliance [28,29,30]. The 5:2 diet is a model of IF consisting of two non-consecutive days per week of 500 kilocalories (kcal) for women and 600 kcal for men; the remaining five days consist of habitual intake [30,31]. Previous studies, mostly with short duration, have reported beneficial effects of IF on clinical outcome measures [27,30]. Studies have shown conflicting results with regards to cardiovascular risk factors such as blood pressure, low-density lipoprotein cholesterol (LDLchol), triglycerides, IR and HbA1c [30,32,33]. A long-term effect of this diet on risk factors for CVD and the feasibility of this diet among patients with T2D needs to be further investigated [27].

In a non-randomized controlled six-month trial of the 5:2 diet with a 12-month follow-up, we have recently shown improvements in markers of insulin secretion and resistance in overweight and obese subjects with and without type 2 diabetes [34]. In a secondary analysis of this trial, we here report results on cardiovascular risk factors including blood pressure, LDLchol, high-density lipoprotein cholesterol (HDLchol), triglycerides, adipokines and inflammatory markers, such as high-sensitivity C-reactive protein (hsCRP) in overweight/obese women and men with and without T2D.

## 2. Results

### 2.1. Baseline Characteristics

Baseline characteristics and age-adjusted *p*-values for the T2D and control group are presented in Table 1. As previously reported [34] the participants with T2D were older than controls but matched with respect to waist, weight and BMI. The participants with T2D had a more severe metabolic profile with significantly higher levels of triglycerides and hsCRP and lower levels of HDLchol and adiponectin than the controls; on the other hand, the controls had significantly higher levels of LDLchol.

### 2.2. Six Months of 5:2 Diet Intervention

In the T2D group, all participants completed the intervention (n = 35, 66% women), whereas in the control group (n = 62, 63% women), four participants were lost to follow-up.

As previously reported [34], in both groups, the 5:2 diet intervention resulted in significant decreases in body weight, −6.3% in the T2D group and −5.0% in the controls, and waist circumference. Moreover, waist circumference decreased significantly more in T2D (*p* = 0.022). In addition, systolic blood pressure (SBP), diastolic blood pressure (DBP) and LDLchol decreased in both groups, and HDLchol and adiponectin increased significantly (Table 2). Participants with T2D showed a significant decrease in alanine aminotransferase (ALT) (*p* ≤ 0.001) and hsCRP (*p* ≤ 0.001). The change in hsCRP in participants with T2D was significantly different from controls (*p* = 0.003), and the change in ALT tended to be different from controls (*p* = 0.051). In the control group, there were significant decreases in triglycerides (*p* = 0.008) and leptin (*p* = 0.003).

For all participants, the change in weight between baseline and six months on the intervention diet correlated positively with changes in triglycerides (*R* = 0.23, *p* = 0.025), LDLchol (*R* = 0.31, *p* = 0.003) and ALT (*R* = 0.26, *p* = 0.01). The change in waist circumference correlated positively with changes in LDLchol (*R* = 0.28, *p* = 0.006) and hsCRP (*R* = 0.27, *p* = 0.012).

### 2.3. Twelve-Month Follow-Up

**Figure 1:**
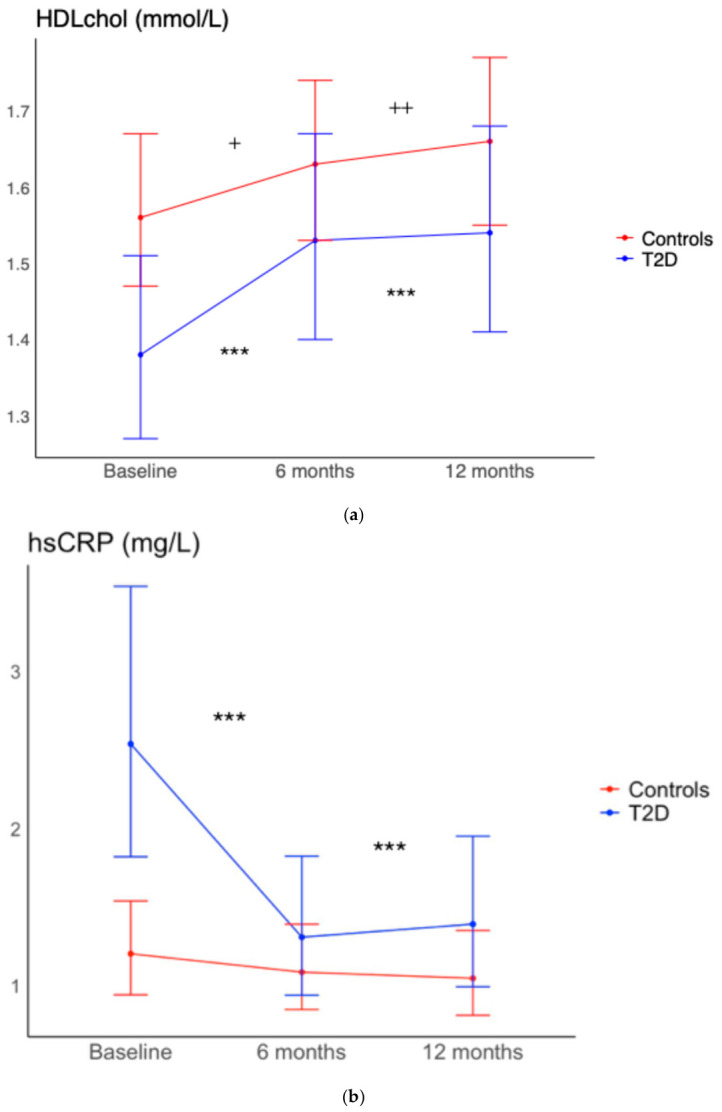
(**a**) Fasting HDLchol and (**b**) hsCRP values at baseline, six-month intervention, and 12-month follow-up for participants with type 2 diabetes (T2D group) and controls, respectively. Within-group differences are represented by *** = *p* < 0.001 for the T2D group and ^+^ = *p* < 0.05, ^++^ = *p* < 0.01 for controls.

Eighty-two (88%) out of the 93 participants who completed the six-month intervention attended the 12-month follow-up: 31 out of 35 participants with T2D and 51 out of 58 in the control group. After the completion of the six-month intervention, body weight had increased on average by 1.90% in the T2D group and 2.18% in the control group at the 12-month follow-up. Still, body weight and waist circumference were significantly reduced compared to baseline, as previously reported [34] (Table 3). Furthermore, both groups had significant increases in HDLchol (Figure 1a) and adiponectin compared to baseline. In addition, the T2D group displayed significant decreases in leptin (*p* = 0.01) and hsCRP (*p* < 0.001) (Figure 1b) and the controls a significant decrease in ALT (*p* = 0.023). The change in hsCRP was significantly different between the groups (*p* < 0.001).

### 2.4. Quality of Life and Physical Activity

Current quality of life (LoL Now) was graded high (8/10) at baseline and showed a moderate but significant further improvement at six months in the whole group (*p* = 0.005) with a tendency to improvement in both T2D and controls (*p* = 0.06). As previously reported, self-reported physical activity (PA) was not significantly influenced by participation in this study [34], no correlations were found between the reported PA and difference in weight, waist circumference or metabolic changes at six months compared to the baseline values in any of the two groups.

## 3. Discussion

The 5:2 diet, a type of IF, resulted in improvements in blood pressure, lipids, adiponectin, leptin and hsCRP, i.e., risk markers of CVD, after six months in both overweight and obese individuals with and without T2D. The positive effects were still present at the 12-month follow-up for HDLchol, adiponectin, leptin and hsCRP in individuals with T2D, and for HDLchol, ALT and adiponectin in controls. The reduction in body weight and waist circumference after the intervention was positively associated with several cardiovascular risk factors.

Systolic and diastolic blood pressure decreased in both groups at six months, but the improvements were not observed at the 12-month follow-up, although there was a tendency for both systolic and diastolic blood pressure to decrease in the control group. Previous IF trials have shown varying results in terms of blood pressure [30,32,35,36] as most studies have only seen an improvement in blood pressure in individuals with pre-existing hypertension [30]. The individuals in the present trial had only a moderately increased blood pressure at baseline which may explain the observed findings. Moreover, it has been suggested that the improvement in blood pressure is mainly through direct effects of the fasting and not the accompanying weight loss [30]. The change in blood pressure may be secondary to decreased IR, as previously reported [34], since the pathological process of IR leads to decreased nitric oxide production, which results in increased vascular resistance and thereby increased blood pressure [37,38].

The decrease in triglyceride levels and LDLchol in the T2D group after the intervention was comparable to the changes reported by Carter et al. [39] investigating the effect of 5:2 diet in a group of T2D patients. The relatively small change in LDLchol observed in the present study may be due to low baseline levels and the fact that 16 out of 35 of the T2D participants were on lipid-lowering medication (statins). The reductions in triglycerides and LDLchol in the controls were less pronounced compared to the findings by Sundfor et al. investigating the effect of the 5:2 diet in obese participants [35]. The difference in magnitude of the lipid changes may be explained by differences in the subjects enrolled and the design of the studies, i.e., younger age of the participants, higher BMI, lower recommended calorie intake (400 kcal per day for women during the fasting days), and larger changes in body weight in the previous trial [35]. In the present study, an increase in HDLchol levels was observed in both groups, contrasting with previous studies on IF [35,39], except for the study by Bhutani et al., where IF was combined with exercise [40]. Comparing CR and IF, Wang et al. found no differences between the diets regarding changes in the lipid profile [33]. The change in HDLchol was still present at the 12-month follow-up in both groups.

The effects of the 5:2 diet on adiponectin and leptin levels in T2D have not, to the best of our knowledge, been reported before. The increase in adiponectin observed in both groups, most prominently in the T2D group, supports the effectiveness of the 5:2 diet, as previous studies have shown that adiponectin is inversely correlated with IR, inflammation, and glucose intolerance [11,12]. The modest decrease in leptin and increase in adiponectin levels in the control group in this study is consistent with previous reports of IF in healthy participants [41]. At the 12-month follow-up, both groups still showed improvements in adiponectin.

The decreases in ALT and hsCRP observed in subjects with T2D, of which the improvement in hsCRP was retained at the 12-month follow-up, are most probably the result of the loss of body and liver fat [2]. Lower levels of hsCRP may reflect a reduction in subclinical inflammation, commonly associated with excessive adipose tissue mass [42]. Individuals with T2D generally exhibit a more adverse metabolic profile than individuals without T2D at a comparable BMI [5,43]. Consequently, the observed decrease in hsCRP suggests an improvement of this more pernicious metabolic profile in participants with T2D. In support, we previously reported from this study [34] that IGFBP-1 levels were increased in the T2D group, which can be explained by reduced insulin secretion and improved hepatic insulin sensitivity due to reduced liver fat accumulation [44]. Holmer et al. have previously shown that the 5:2 diet was effective in reducing liver steatosis [2].

Glycemic control is a known cardiovascular risk factor. In the T2D group HbA1c and fasting glucose were improved at six months, with no changes in controls who had normal levels at baseline [34]. At the 12-month follow-up, six months after the end of the intervention, fasting glucose compared to baseline levels was improved in both groups; however, there was no significant decrease in HbA1c, which in the T2D group was low (mean 46.1 mmol/mol) at 12 months [34].

Only three of the 35 subjects with T2D were for more than one year on low-dose GLP1-RA (Liraglutide), which they continued during this study. Before participation in this study, they had been weight-stable for at least a year, while they lost weight during the study period as did the other 32 subjects. Thus, medication with Liraglutide most probably did not impact the results.

One limitation of this study could be the lack of a comparison with another dietary intervention, such as daily CR. However, the purpose of our study was to compare the effect of the 5:2 diet in subjects with and without T2D and should be regarded as a feasibility trial showing that the 5:2 diet has the potential to improve anthropometric and metabolic status. Due to age differences between the groups, age adjustments were performed. To account for the drop-out rate, especially at the 12-month follow-up, ITT analyses were performed. We acknowledge that no objective measurements of daily energy intake and physical activity were obtained, as the parameters were only assessed through questionnaires; in the absence of objective markers, we cannot fully exclude misreporting or imperfect compliance. This fact, and with no comparison with CR, implies that this study cannot guarantee that the effects solely were due to the 5:2 diet and fasting; however, there was an observed significant weight loss and improved metabolic status. Participants were recruited through public advertisement and responding to a questionnaire. This recruitment strategy favors individuals who are already motivated to lose weight; all had a history of long-standing overweight and previous, unsuccessful, attempts to reduce their weight. However, it should be noted that essentially anyone who volunteers for a weight-loss intervention is motivated to some degree, and our cohort and most cohorts of dietary studies are unlikely to be fully representative of the broader population with overweight or type 2 diabetes. This self-selection may limit the generalizability of our findings. In addition, this study is a non-randomized, controlled design. This raises the possibility that the observed adherence, weight loss, and metabolic improvements partly reflect pre-existing factors of the participants rather than the effect of the 5:2 diet. Despite adjustment for age, confounding from such unmeasured factors cannot be excluded. Taken together these considerations limit the generalizability of our findings, which may not extend to less motivated or unselected populations. Consequently, validation in randomized controlled trials is warranted to confirm these findings. Future studies should compare CR to the 5:2 diet and could be divided into groups of overweight or obese participants with and without T2D.

The strengths of this study include the duration of the intervention and the 12-month follow-up and the low drop-out rate during both the six-month intervention and the 12-month follow-up. Most previous studies have had a shorter duration, few being longer than three months with no follow-up [28,30,33]. Moreover, most dietary interventions are characterized by high drop-out rates largely due to participant attrition [20]. An additional strength is that the percentages of females were similar in the two study groups.

We have previously reported that the 5:2 diet is effective and feasible to follow, resulting in weight reduction of 5–6% and improved insulin sensitivity [34]. Here we report that cardiovascular risk factors were improved after six months on the 5:2 diet in overweight or obese individuals with and without T2D. These improvements have been reported before but the effectiveness in obese or overweight subjects with and without T2D have not been compared previously. Sustained improvements were also seen for HDLchol and adiponectin for both groups at the 12-month follow-up. Given the inconsistent findings from previous IF studies regarding cardiovascular risk factors, the present study provides valuable insights that the 5:2 diet may improve cardiometabolic risk markers and overall health in overweight and obese individuals, both with and without type 2 diabetes. Notably, individuals with type 2 diabetes appeared to derive greater metabolic benefits from the intervention compared with controls.

## 4. Materials and Methods

### 4.1. Study Design

This study is a non-randomized controlled trial evaluating the impact of the 5:2 diet on markers of insulin secretion and insulin sensitivity as primary outcomes in overweight and obese individuals, both with and without T2D and cardiovascular risk markers as secondary outcomes. The intervention period lasted six months, followed by a 12-month post-intervention follow-up. This study complied with Swedish regulations for human research and was approved by the Regional Ethical Review Board in Stockholm (Dnr: 2013/1618-31/3). It was registered at ClinicalTrials.gov (Identifier: NCT02450097).

### 4.2. Eligibility Criteria

Inclusion criteria: Adults aged ≥ 18 years, with or without T2D, a BMI between 25 and 37 kg/m^2^, and a waist circumference > 80 cm for women or >94 cm for men.

Exclusion criteria: BMI < 25 kg/m^2^, waist circumference ≤ 80 cm for women or ≤94 cm for men, treatment with insulin or sulfonylureas, any condition in which weight loss was contraindicated, chronic kidney disease (eGFR < 30 mL/min), pregnancy or breastfeeding, competitive athletic activity, history of eating disorders, a current cancer diagnosis, or participation in another ongoing study.

### 4.3. Subjects

Participants were recruited through public advertisements and initially screened via a digital questionnaire. All participants provided written informed consent. Screening was conducted in groups of 10–12 individuals. The recruitment goal was 40 T2D participants and 80 normoglycemic overweight/obese controls BMI- and waist circumference-matched. Due to prolonged recruitment time, enrollment was stopped after 104 individuals (37 with T2D, 67 controls) completed screening. Seven individuals were excluded after screening, one not meeting the criteria and six discontinuing due to; pregnancy (n = 1), a new serious diagnosis (n = 1), inability to follow the diet (n = 2), and unknown reasons (n = 2).

A total of 97 participants entered the six-month intervention (35 with T2D and 62 controls). Among them, 39 (63%) controls and 23 (66%) participants with T2D were women. Three T2D participants discontinued sulfonylurea treatment prior to enrollment. Of the 35 participants with T2D, 13 were treated with solely diet, 10 with metformin monotherapy, eight with metformin plus a DPP-4 inhibitor, two with metformin plus low-dose GLP-1RA (Liraglutide), one with metformin plus a sodium-glucose cotransporter 2 (SGLT2) inhibitor, and one with combination therapy including metformin, SGLT2 inhibitor, and GLP-1RA at baseline.

### 4.4. Procedure

This study was conducted between 2013 and 2015 at the Department of Endocrinology, Metabolism, and Diabetes, Karolinska University Hospital, and followed Good Clinical Practice (GCP) standards.

Participants attended clinic visits before the start of the intervention and at 6, 12, and 24 weeks (final visit). After the intervention, they were encouraged to attend a 12-month follow-up visit without further dietary instructions; during this period, they were only recommended to record their eating habits and weigh themselves weekly. A medical doctor conducted the baseline examination; subsequent visits were conducted by a nurse.

At baseline, three months, and six months, assessments were performed after an overnight fast. These included blood pressure (measured twice after 10 min of seated rest; the lower value was used), blood sampling, waist circumference measured midway between the lower rib and iliac crest, and body composition (weight, total body fat, trunk fat) using bioimpedance analysis (Tanita BC-418 Tanita body composition analyzer BC-418, Tanita Corporation, Tokyo, Japan). BMI was calculated as weight (kg) divided by height squared (m^2^). At each visit, participants submitted completed questionnaires on PA, self-rated health, quality of life, and fasting-day food diaries.

### 4.5. Dietary Intervention

Before starting the intervention, each cohort of 10–14 participants attended a group information session led by one or two physicians, two nurses, and a dietitian. The session covered study procedures, background information, and a lecture on the Nordic Nutrition Recommendations, including information on the Mediterranean and Nordic dietary patterns.

Participants were instructed to follow the 5:2 dietary regimen, consisting of two non-consecutive fasting days per week. On the remaining five days, participants were instructed to eat as they normally would and were recommended to follow Mediterranean or Nordic dietary principles.

On fasting days, energy intake was limited to 500 kcal/day for women and 600 kcal/day for men, with a minimum protein intake of 20 g/day. Calorie-free or very low-calorie beverages (e.g., water, coffee, tea) were allowed without restriction. Participants received 20 recipes for fasting-day meals (100–400 kcal each), high in fiber and protein and low in fat, designed to optimize satiety, minimize preparation time, and provide good nutritional balance. Meal options included vegetarian, fish, red meat, poultry, whole grains, dairy, legumes, eggs, vegetables, and fruits. Choosing 2–3 meals per fasting day from these menus would result in an estimated 20–25% weekly energy reduction. The choice of menus for the two fasting days was reported every week during the six-month intervention. Participants did not report food intake during non-fasting days. Adherence to the 5:2 diet was assessed by questionnaire reports on the choice of menus collected at every visit and through discussion. Participants also rated hunger, satiety, and adherence capability on Visual Analog Scales (VAS) each fasting night at approximately 10 p.m.

### 4.6. Questionnaires

No specific recommendations were given regarding PA. PA was assessed using a validated questionnaire addressing four domains: PA at work, transportation-related PA, leisure-time PA, and total PA [45,46].

Self-rated health (SRH) was measured using items on overall perceived health and comparisons with peers of the same age, as well as Cantril’s Ladder of Life. Participants rated current, past-year, and anticipated quality of life on a 10-point scale from 1 (“Worst imaginable life”) to 10 (“Best possible life”) [47].

### 4.7. Laboratory Analyses

All blood samples were collected fasting. Triglycerides, total cholesterol, HDLchol, and ALT were measured using enzymatic photometric assays (Cobas c701, Roche Diagnostics International Ltd., Rotkreuz, Switzerland) at the Karolinska University Hospital laboratory. Fasting LDLchol was calculated according to the Friedewald formula: LDLchol = total cholesterol − HDLchol − 0.45 × triglycerides [48]. hsCRP levels were determined by immunoturbidimetric assay (Cobas c502, Roche Diagnostics International Ltd., Rotkreuz, Switzerland). Leptin and adiponectin concentrations were analyzed using a commercial radioimmunoassay kit (EMD Millipore Corporation, Billerica, MA, USA). For leptin, inter-assay precision was 3.6–6.2% and intra-assay precision was 3.4–8.3%. For adiponectin, inter-assay precision was 6.9–9.3% and intra-assay precision was 1.8–6.2%.

### 4.8. Outcomes

The outcomes examined here are secondary endpoints from the trial, related to cardiovascular risk in overweight and obese adults with and without T2D.

### 4.9. Statistics

RStudio (Version 1.4.1106) was used for all analyses. The Shapiro–Wilk test, skewness evaluation, histogram and QQ plot were used to test whether the current variables met the requirement for normal distribution at baseline. Independent samples *t*-tests were used to determine baseline differences between the two study groups’ weight, BMI waist circumference, SBP and DBP. Baseline comparisons for triglycerides, HDLchol, LDLchol, ALT, adiponectin, leptin and hsCRP were age-adjusted using a linear model with age as a covariate.

Mixed-model ANOVA with factors Group, Time, and Group × Time interaction was used to analyze within- and between-group differences, following the intention-to-treat principle. Results in Table 2 and Table 3 are age-adjusted.

Variables with positive skewness were log-transformed prior to mixed-model analysis, and results are presented as antilog values in Table 2 and Table 3. Graphical presentations of HDLchol and hsCRP values are geometric means with 95% confidence intervals.

Because the variable age violated the assumption of equal variances, mean differences were calculated using the Satterthwaite–Welch correction. In Table 1, normally distributed anthropometric data are shown as mean ± SD; non-normally distributed variables are shown as median (IQR). Results in Table 2 and Table 3 are presented as means with 95% confidence intervals. A *p*-value < 0.05 was considered statistically significant.

## Figures and Tables

**Table 1:** Baseline characteristics for the T2D and control group.

	T2D	Controls	*p*-Value
No. (% women)	35 (66%)	62 (63%)	
Age (year)	66.63 ± 8.11	57.98 ± 11.76	**<0.001**
Weight (kg) *	82.37 ± 15.26	86.35 ± 15.61	0.23
BMI (kg/m^2^) *	28.55 ± 3.46	28.85 ± 4.04	0.71
Waist (cm) *	103.83 ± 10.50	100.87 ± 10.83	0.19
SBP (mmHg)	137.1 ± 16.39	133.7 ± 16.77	0.33
DBP (mmHg)	79.93 ± 9.35	82.53 ± 9.21	0.19
Triglycerides (mmol/L)	1.40 (1.10–1.70)	1.00 (0.76–1.48)	**<0.001**
HDLchol (mmol/L)	1.40 (1.20–1.55)	1.60 (1.30–1.80)	**0.029**
LDLchol (mmol/L)	2.60 (2.03–3.70)	3.65 (3.10–4.20)	**<0.001**
ALT (µkat/L)	0.42 (0.32–0.52)	0.42 (0.30–0.57)	0.086
Adiponectin (µg/mL)	9.0 (7.2–14.0)	13.1 (10.5–18.5)	**<0.001**
Leptin (ng/mL)	26.9 (13.7–36.4)	25.4 (13.6–36.6)	0.11
hsCRP (mg/L)	2.40 (1.40–4.30)	1.20 (0.61–2.48)	**0.002**

Values presented as mean ± SD or as median and interquartile range (25th–75th). Significant results presented in bold. ALT = alanine aminotransferase, hsCRP = high-sensitivity C-reactive protein, DBP = diastolic blood pressure, HDLchol = high-density lipoprotein cholesterol, LDLchol = low-density lipoprotein cholesterol, SBP = systolic blood pressure. * Denotes previously published results [34]. Triglycerides, HDLchol, LDLchol, ALT, Adiponectin, Leptin and hsCRP *p*-values are based upon age-adjusted analyses.

**Table 2:** Anthropometric and metabolic variables before and six months after intervention in the T2D group and controls.

	T2D (66% Females)			Controls (63% Females)				
	Baseline (n = 35)	6 Months (n = 35)	*p*-Value	Baseline (n = 62)	6 Months (n = 58)	*p*-Value	Difference Diabetics–Controls	*p*-Value
Weight (kg) *	84.9 (79.8–89.9)	79.6 (74.5–84.7)	**<0.001**	84.6 (80.8–88.4)	80.4 (76.6–84.3)	**<0.001**	−1.05 (−2.37; 0.26)	0.12
BMI (kg/m^2^) *	29.2 (27.9–30.5)	27.4 (26.1–28.7)	**<0.001**	28.4 (27.5–29.4)	27.0 (26.0–27.9)	**<0.001**	−0.39 (−0.83; 0.05)	0.08
Waist (cm) *	104 (100–108)	99 (95–103)	**<0.001**	101 (98–104)	97 (94–100)	**<0.001**	−1.7 (−3.07; −0.25)	**0.02**
SBP (mmHg)	135 (130–140)	128 (123–133)	**0.007**	135 (132–139)	131 (127–135)	**0.036**	−2.52 (−7.98; 2.95)	0.36
DBP (mmHg)	79.9 (76.6–83.1)	76.5 (73.2–79.7)	**0.014**	82.6 (80.2–85.0)	79.5 (77.1–81.9)	**0.003**	−0.38 (−3.35; 2.61)	0.80
Triglycerides (mmol/L) ^L^	1.48 (1.27–1.74)	1.31 (1.12–1.53)	0.13	1.05 (0.93–1.18)	0.90 (0.80–1.02)	**0.008**	1.03 (0.87; 1.20)	0.76
HDLchol (mmol/L) ^L^	1.38 (1.27–1.51)	1.53 (1.40–1.67)	**<0.001**	1.56 (1.46–1.67)	1.63 (1.53–1.74)	**0.049**	1.06 (1.00; 1.12)	0.069
LDLchol (mmol/L)	2.85 (2.52–3.17)	2.52 (2.20–2.85)	**0.013**	3.69 (3.44–3.93)	3.40 (3.16–3.65)	**0.003**	−0.04 (−0.32; 0.24)	0.78
ALT (µkat/L) ^L^	0.47 (0.40–0.56)	0.37 (0.31–0.44)	**0.001**	0.40 (0.35–0.45)	0.37 (0.32–0.42)	0.19	0.85 (0.73; 1.00)	0.051
Adiponectin (µg/mL) ^L^	9.2 (7.9–10.7)	10.1 (8.7–11.7)	**0.014**	13.9 (12.4–15.5)	14.9 (13.3–16.8)	**0.007**	1.02 (0.94; 1.10)	0.71
Leptin (ng/mL) ^L^	28.5 (20.9–38.9)	24.0 (17.6–32.5)	0.21	19.7 (15.5–24.8)	15.0 (11.8–18.9)	**0.003**	1.10 (0.85; 1.43)	0.48
hsCRP (mg/L) ^L^	2.54 (1.82–3.54)	1.31 (0.94–1.82)	**<0.001**	1.20 (0.94–1.54)	1.09 (0.85–1.40)	0.50	0.57 (0.42; 0.77)	**<0.001**

Values presented as mean (95% CI). Significant results presented in bold. ALT = alanine aminotransferase, ANOVA = analysis of variance, hsCRP = high-sensitivity C-reactive protein, DBP = diastolic blood pressure, HDLchol = high-density lipoprotein cholesterol, LDLchol = low-density lipoprotein cholesterol, SBP = systolic blood pressure. * Denotes previously published results [34]. ^L^ Denotes antilog values and *p*-values from analyses with log-transformed data. Analyses performed with Mixed Model ANOVA and based on ITT and age adjustment. *p*-values in the rightmost column represent differences in change between participants with and without type 2 diabetes; all other *p*-values represent intergroup differences.

**Table 3:** Anthropometric and metabolic variables before and at 12-month follow-up in the T2D group and controls.

	T2D (66% Females)			Controls (62% Females)				
	Baseline (n = 35)	12 Months (n = 31)	*p*-Value	Baseline (n = 62)	12 Months (n = 51)	*p*-Value	Difference Diabetics–Controls	*p*-Value
Weight (kg) *	84.9 (79.8–89.9)	81.0 (75.9–86.1)	**<0.001**	84.6 (80.8–88.4)	81.9 (78.1–85.8)	**<0.001**	−1.19 (−2.57; 0.20)	0.09
BMI (kg/m^2^) *	29.2 (27.9–30.5)	27.8 (26.5–29.1)	**<0.001**	28.4 (27.5–29.4)	27.5 (26.5–28.5)	**<0.001**	−0.45 (−0.91; 0.01)	0.057
Waist (cm) *	104 (100–108)	100 (96–104)	**<0.001**	101 (98–104)	97 (94–100)	**<0.001**	−1.0 (−2.5; 0.5)	0.20
SBP (mmHg)	135 (130–140)	134 (129–139)	0.89	135 (132–139)	131 (127–135)	0.08	2.84 (−2.91; 8.58)	0.33
DBP (mmHg)	79.9 (76.6–83.1)	77.5 (74.2–80.8)	0.14	82.6 (80.2–85.0)	80.3 (77.8–82.8)	0.054	−0.12 (−3.23; 3.00)	0.94
Triglycerides (mmol/L) ^L^	1.48 (1.27–1.74)	1.37 (1.17–1.62)	0.49	1.05 (0.93–1.18)	1.07 (0.95–1.21)	0.91	0.91 (0.76; 1.07)	0.25
HDLchol (mmol/L) ^L^	1.38 (1.27–1.51)	1.54 (1.41–1.68)	**<0.001**	1.56 (1.46–1.67)	1.66 (1.55–1.77)	**0.007**	1.05 (0.98; 1.12)	0.15
LDLchol (mmol/L)	2.85 (2.52–3.17)	2.81 (2.47–3.14)	0.95	3.69 (3.44–3.93)	3.47 (3.22–3.72)	0.051	0.18 (−0.12; 0.47)	0.23
ALT (µkat/L) ^L^	0.47 (0.40–0.56)	0.44 (0.37–0.52)	0.50	0.40 (0.35–0.45)	0.35 (0.31–0.40)	**0.023**	1.06 (0.90; 1.26)	0.47
Adiponectin (µg/mL) ^L^	9.2 (7.9–10.7)	10.0 (8.6–11.6)	**0.049**	13.9 (12.4–15.5)	14.9 (13.3–16.8)	**0.013**	1.00 (0.92; 1.09)	0.92
Leptin (ng/mL) ^L^	28.5 (20.9–38.9)	20.7 (15.0–28.5)	**0.01**	19.7 (15.5–24.8)	18.2 (14.2–23.1)	0.63	0.79 (0.60; 1.03)	0.09
hsCRP (mg/L) ^L^	2.54 (1.82–3.54)	1.39 (0.99–1.95)	**<0.001**	1.20 (0.94–1.54)	1.05 (0.81–1.35)	0.32	0.63 (0.46; 0.86)	**0.004**

Values presented as mean (95% CI). Significant results presented in bold. ALT = alanine aminotransferase, ANOVA = analysis of variance, hsCRP = high-sensitivity C-reactive protein, DBP = diastolic blood pressure, HDLchol = high-density lipoprotein cholesterol, LDLchol = low-density lipoprotein cholesterol, SBP = systolic blood pressure. * Denotes previously published results [34]. ^L^ Denotes antilog values and *p*-values from analyses with log-transformed data. Analyses performed with Mixed Model ANOVA and based on ITT and age adjustment. *p*-values in the rightmost column represent differences in change between participants with and without type 2 diabetes; all other *p*-values represent intergroup differences.

## Data Availability

The raw data supporting the conclusions of this article will be made available by the authors on reasonable request.
